# Deep Learning-Based Automated Cell Detection-Facilitated Meat Quality Evaluation

**DOI:** 10.3390/foods13142270

**Published:** 2024-07-18

**Authors:** Hui Zheng, Nan Zhao, Saifei Xu, Jin He, Ricardo Ospina, Zhengjun Qiu, Yufei Liu

**Affiliations:** 1 College of Biosystems Engineering and Food Science, Zhejiang University, Hangzhou 310058, China; huizheng@zju.edu.cn (H.Z.); 21913012@zju.edu.cn (N.Z.); zjqiu@zju.edu.cn (Z.Q.); 2 College of Animal Sciences, Zhejiang University, Hangzhou 310058, China; 3180100618@zju.edu.cn (S.X.); hejin@zju.edu.cn (J.H.); 3 Research Faculty of Agriculture, Hokkaido University, Sapporo 060-8589, Japan; rospina@agr.hokudai.ac.jp

**Keywords:** cell counting, cell classification, meat quality, deep learning

## Abstract

Meat consumption is increasing globally. The safety and quality of meat are considered important issues for human health. During evaluations of meat quality and freshness, microbiological parameters are often analyzed. Counts of indicator cells can provide important references for meat quality. In order to eliminate the error of manual operation and improve detection efficiency, this paper proposed a Convolutional Neural Network (CNN) with a backbone called Detect-Cells-Rapidly-Net (DCRNet), which can identify and count stained cells automatically. The DCRNet replaces the single channel of residual blocks with the aggregated residual blocks to learn more features with fewer parameters. The DCRNet combines the deformable convolution network to fit flexible shapes of stained animal cells. The proposed CNN with DCRNet is self-adaptive to different resolutions of images. The experimental results indicate that the proposed CNN with DCRNet achieves an Average Precision of 81.2% and is better than traditional neural networks for this task. The difference between the results of the proposed method and manual counting is less than 0.5% of the total number of cells. The results indicate that DCRNet is a promising solution for cell detection and can be equipped in future meat quality monitoring systems.

## 1. Introduction

Meat consumption is increasing globally [1,2] and the scale of animal husbandry continues to expand [3,4]. Meanwhile, adverse environment and unprofessional management during animal breeding, storage and transportation can make it hard to ensure meat safety and quality. It is crucial in the meat industry to accurately assess meat quality attributes by introducing advanced techniques [5,6]. Some researchers designed a spectroscopy method, electronic nose or electronic tongue to evaluate meat quality [7,8]. However, most researchers perform microbiological analysis in parallel. Researchers use microbial cell number as a measure of meat spoilage [9,10,11]. This means that counting of microbial cells is still the formal method to evaluate the quality and the freshness of meat. Currently, the counts of cells mainly depend on manual observation under the microscope using a blood cell counting plate for suspended cells and histological sections [12,13], which is often expensive and time-consuming and requires qualified personnel [14]. Therefore, with the development of computer vision algorithms, using macroscopic and microscopic images of meat to detect meat spoilage automatically has become a research hotspot [15,16,17]. The counting and classification of cells can identify disease markers, quantify cell status and evaluate meat quality [18,19,20]. The proportion or number of stained cells can be an important reference for the measurement of meat quality. Efficient and accurate cell recognition and counting can improve the efficiency of meat quality testing.

There have already been some automated cell counting technologies developed [21,22]. The Coulter Counter [23] is an instrument that can count the suspended cells when the cells go through a pinhole. Flow Cytometry [24] is a laser-based technique used to detect and analyze specific components or properties of cells in liquid suspension. However, both instruments are limited to using a single cell suspension rather than adherent cells. The Coulter Counter can only identify cells based on size instead of color. As for the Flow Cytometer, many factors and parameters like dyeing schemes must be considered before the counting process and the costs of the instruments and sample preparation are high. Therefore, it would not be user-friendly nor easy for a newcomer to utilize this technology. The Countess™ 3 Automated Cell Counter (ThermoFisher Co., Ltd., Carlsbad, CA, USA) is equipped with a deep learning algorithm. It can count different types of cells and analyze their viability. However, this instrument is limited to treating suspended cell solutions and cannot be used for histological sections. Additionally, deformed and clustered cells bring a huge challenge to high-precision automated cell recognition.

This paper presents a new algorithm to better characterize cell nuclei staining in animal cell sections in an automated and user-friendly manner. This algorithm based on deep learning can automatically identify and count stained cell nuclei with high sensitivity, which can liberate people from tedious and repetitive labor. As a result, researchers can direct their energy toward more important scientific research. In addition, the goal of this study is to reduce or even eliminate the errors in the results created by different individuals, instruments and environments during experiments.

## 2. Materials and Methods

To categorize the cells in the images as stained or unstained, two processing steps are necessary. First, all of the cells in the images must be detected. Second, the detected cells need to be analyzed to determine if they are stained or unstained based on their color.

Accomplishing these two steps requires a robust dataset and an effective detection and classification algorithm. Therefore, this article presents a dataset created by the authors and introduces a novel backbone for a CNN.

### 2.1. Dataset

An appropriate and comprehensive dataset is the key to obtaining a reliable model. In order to be more targeted, the dataset should contain corresponding data instead of universal data. In this study, an original corresponding dataset is created. Chinese experimental mini-pigs (CEMPs) were established through full-sib inbreeding and negative selection of Xiang pigs. The samples came from CEMP kidney cells with polycystic kidney disease. Kidneys from CEMPs were harvested, minced and fixed in a 10% neutral buffered formalin solution. Then, the samples were dehydrated and embedded in paraffin. The nuclei were stained with Ki-67 [25]. The cell images were obtained by using a digital microscope camera (Olympus, DP70, Tokyo, Japan). Considering that different operators would adopt different parameters for imaging, several sets of common parameters were adopted (exposure is 1/1400, plotting scale is 4080 × 3072, variable resolution, as shown in Table 1) to obtain cell images which cover the most common imaging conditions and enrich the dataset. The microscope was set to capture cell images with resolutions of 3840 × 2880 and 2560 × 1920. Finally, a set of 435 images was captured, which consisted of 225 images in 3840 × 2880 and 210 images in 2560 × 1920. These images captured by the digital microscope camera were defined as original cell images. The original cell images with different resolutions were divided into a training set, a validation set and a test set, respectively, according to the ratio of 6:3:1.

Using data augmentation technology is a common method for deep learning to avoid form overfitting [26]. There were two steps to accomplish data augmentation in this study. The first step was that the images were flipped both horizontally and vertically. As for an individual cell, its orientation changes randomly due to its movement and the extrusion of other cells. Flipping the cell images allows the dataset to cover more cells’ orientation information and helps the deep learning model to learn more details of the orientation features. After flipping, the second step was to cut all of the images into smaller sizes. In real experiments, different experiment operators use different plotting scales. On different plotting scales, the cells’ number of pixels and the ratio of the cells’ pixels to the entire image’s pixels are different. Hence, it is a multi-scale task for the deep learning model to detect cells at different plotting scales. In order to make the model more reliable, it was necessary to enrich the multi-scale information in the dataset. Cutting cell images into several smaller sizes is an effective method. In one image, the number of pixels required to display one cell is relatively fixed. If the resolution of the cell images changes, the ratio of the cell’s size to the image’s size will also change, which means the relative size of a cell will change accordingly. Thus, cutting cell images into smaller sizes produces multi-scale objects and improves the model’s performance in multi-scale detection tasks. In order to make full use of the information in the images, the resolution of the original images should be an integral multiple of the small-sized images’ resolution. Meanwhile, the resolutions of small-sized images should be typical and should be divisible by mainstream resolutions. Therefore, each original cell image in the dataset was cut into small-sized images with resolutions of 320 × 240, 640 × 480 and 1280 × 960. The number of images in each set is shown in Table 2.

**Figure 1:**
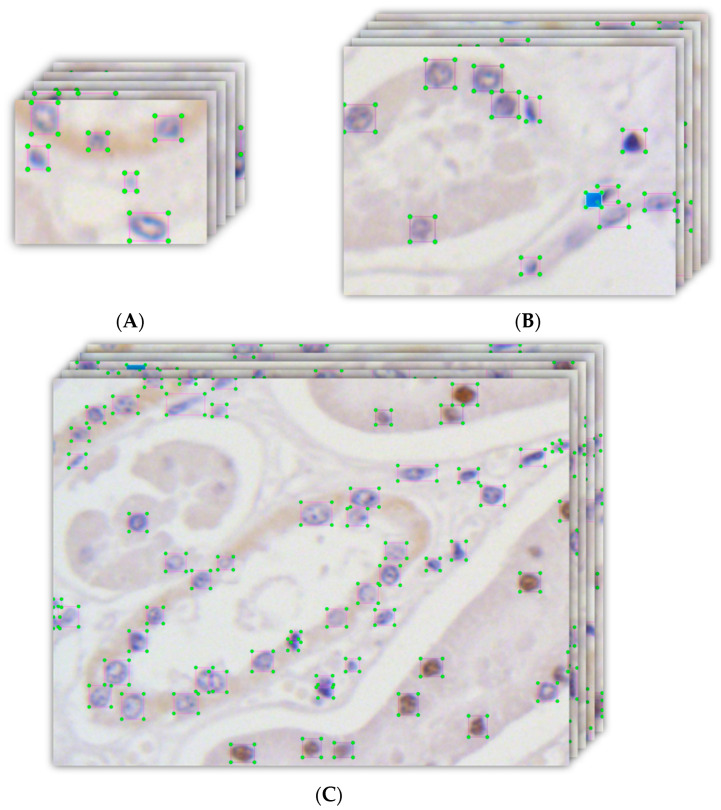
Parts of the labeled images in the dataset. The resolution of the images in (**A**) is 320 × 240, in (**B**), it is 640 × 480, and in (**C**), it is 1280 × 960. The labeled blue cells are unstained, and the brown cells are stained with either Ki-67 antibody or TUNEL kit.

Labeling these images was another essential operation. In the obtained images, there were two classes of cells: blue stained cells and brown unstained cells. An image labeling tool (LabelImg v1.8.5) was used to complete the labeling task in this study. A part of the labeled dataset of cells is shown in Figure 1, where the rectangles are the ground truths.

### 2.2. Cell Detection Algorithm

**Figure 2:**
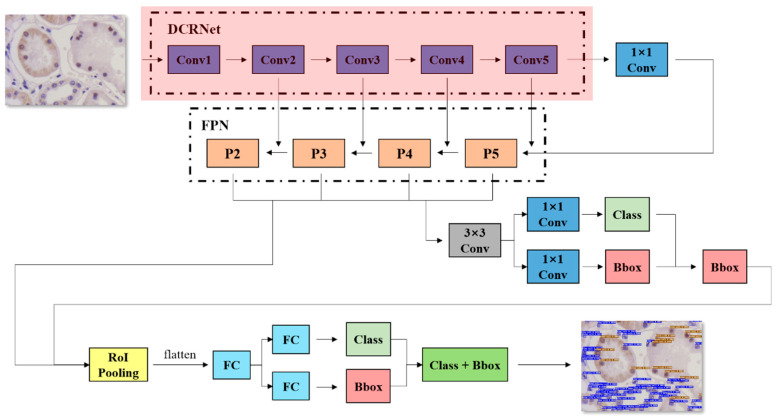
The overall structure of the detection network with DCRNet.

In order to detect and recognize cells using deep learning methods, it is important to employ a reliable object detection-based algorithm. CNN modules can build an effective detection network which is able to draw bounding boxes and classify cells. Upon these considerations, this work introduces an original neural network based on Faster R-CNN [27]. The novel backbone DCRNet based on ResNet-101 takes inspiration from the design of Faster R-CNN. The network’s structure is shown in Figure 2.

Firstly, the image is transformed into a fixed size (1200 × 1800 in this study). The image with a fixed size will then enter a Feature Pyramid Network (FPN). ReLU (Rectified linear unit) is used to activate the convolutional layers [28]. The activated layers will be handled by pooling layers to generate the feature maps. The Region Proposal Network (RPN) uses the feature maps to generate the positive anchors and box regression. It will then produce the region proposals and transport them to the Region-of-Interest (RoI) pooling layers. The RoI pooling layers are able to obtain proposal features and transport them to the classification network.

Additional effective methods were applied to make the model converge better in the backbone of DCRNet. Faster R-CNN uses the VGG-16 to extract features, which is unfit for extracting features of small-sized objects [29]. Therefore, the authors adopted ResNet-101 instead of the VGG-16 [30]. ResNet-101 improved the accuracy by widening and deepening the network, but it caused hyperparameter explosion and an increase in computation cost [31]. Therefore, this work aimed to improve the detection accuracy while keeping the floating-point arithmetic within an acceptable range. Inspired by Inception-V4, DCRNet used the strategy of split–transform–merge [32]. Authors replaced the original residual block with a 64-path aggregated residual block to perform the convolution process. As a result, it was possible to learn more features of small objects like cells. Meanwhile, the modified aggregated residual block with fewer parameters has a similar computational complexity to the original ResNet block.

The down-sampling block was also adjusted. In ResNet, down-sampling is completed by a 1 × 1 convolutional kernel with a stride of 2, which will lose the information of some grids of the input feature map. Those grids that are ignored may contain important information. Especially for small targets like cells, each grid may contain key information about whether they are stained. Therefore, in DCRNet, the 1 × 1 convolutional layer is replaced by two parts. The authors used an average pooling layer with a stride of 2 and a 1 × 1 convolutional layer with a stride of 1 to make sure that there is no information loss and that the dimension of the output stays the same. These adjustments practically improved the accuracy.

The convolutional kernels were also modified. Due to the squeezing among the tubular epithelial cells and interstitial cells, they may take on various shapes (spindly, fusiform) rather than being round or oval. How to extract the feature of daedal cells’ shapes and make it contributive to model training is a challenge. A traditional CNN usually relies on extending the dataset or using algorithms like Scale-invariant feature transform (SIFT) to solve relevant problems [33,34]. Instead, this study tried to solve these problems by modifying the backbone. The ‘shape’ of an object is defined as the edge lines between the object and the background. Dynamic convolution can adjust the parameters in the kernel automatically, which is effective for identifying the edge features of cells. In order to use the cells’ ‘shape’ feature better, deformable convolution networks (DCNs) were adopted [35]. They are represented by Equation (1):

(1) $$Y\left(P_{0}\right)=\sum_{P_{n}\in R}w\left(P_{n}\right)\cdot X(P_{0}+P_{n}+∆P_{n}),$$

**Figure 3:**
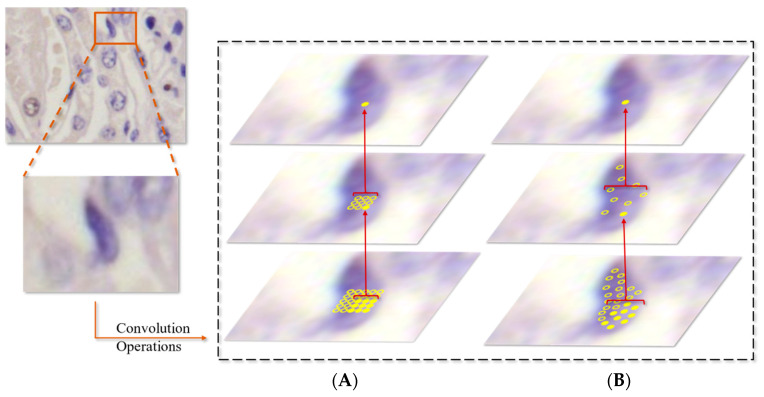
An illustration of (**A**) a fixed receptive field of a fixed convolutional kernel in standard convolution and (**B**) an adaptive receptive field of a deformable kernel in deformable convolution.

In Equation (1), R is a regular grid on the input feature map X; w is the sampled values’ weights; $P_{0}$ is the location in the feature map Y; $P_{n}$ is the location in the regular grid R. In line with DCNs, the offset parameters of the kernel can be trained, as shown in Figure 3.

### 2.3. Ethical Statement

All of the procedures were conducted according to the guidelines developed by the China Council on Animal Care and Protocol and were approved by China Agricultural University (No. SKLAB-2012-04-03).

## 3. Results

### 3.1. Model Performance Evaluation

Effective training is an important step to develop a reliable model. To make the model converge more quickly, the network was trained on the COCO dataset for 160 epochs in advance. The output weights were saved for further training.

**Figure 4:**
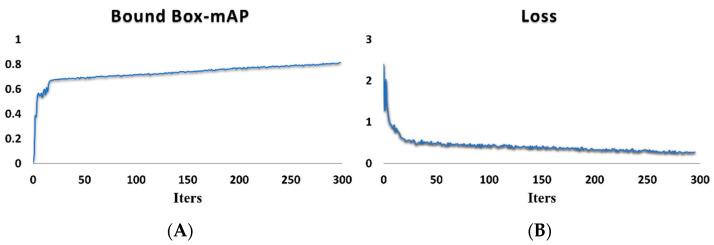
The trends in (**A**) the mean AP (mAP) for predicted bounding boxes and (**B**) losses in training of DCRNet.

Low-resolution (at a resolution of 640 × 480 or lower) images were trained for 30 epochs at first. Cells in low-resolution images have larger relative sizes compared to the image. This trick makes the model learn more information about the cells. Therefore, training low-resolution images firstly helped the model learn more features about small objects and converge faster. Then, the model went on to be trained on the complete training set for 270 epochs. The batch size was set to 4. The loss was recorded every five iterations. The training process is shown in Figure 4. The Average Precision (AP) in Figure 4A is an indicator to measure how many samples are correctly positive in the total predicted samples by using bounding boxes.

In order to see the advantage of DCRNet, authors have conducted the ablation experiments, whose the results of which are shown in Table 3. AP50 means the Average Precision (AP) at the Intersection over Union (IoU) at 50%. AP75 means the AP at the IoU at 75%. APS shows the AP for small objects whose pixels are less than 32 × 32 pixels. APM is for middle-sized objects whose pixels are between 32 × 32 and 96 × 96, and APL is for large objects whose pixels are more than 96 × 96 in size. For a more intuitive display, the results in Table 3 are magnified by 100.

The authors trained some mainstream object detection models in the same way and compared them with DCRNet. The prediction results of the trained models are shown in Table 4. Based on the results, we believe that the DCRNet-based detection model is more accurate than other mainstream models, including Faster R-CNN, SSD and Yolov3. With DCRNet, the Faster R-CNN model produced better prediction results with an acceptable processing speed. In line with the purpose of this study, DCRNet has apparent advantages in detecting cells, based on the column APS in Table 4. In conclusion, the proposed DCRNet-based detection model is more balanced while performing better.

### 3.2. Practical Experiment

A practical experiment was conducted to validate the accuracy and reliability of DCRNet. In the practical experiment, a pathology experimentalist was asked to count cells manually for comparison. The pathology experimentalist and DCRNet conducted the counting operation on the same picture; the counting results of DCRNet and manual operation are shown in Table 5.

**Figure 5:**
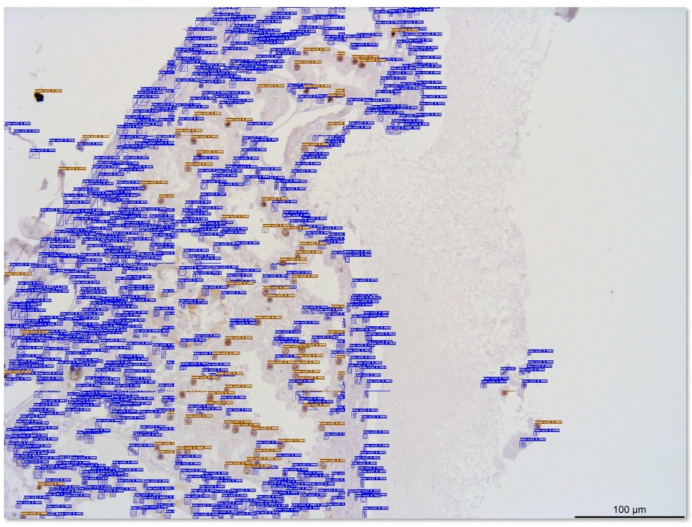
A typical result of cell detection and recognition in this study.

In addition, it is worth paying attention to the detailed level of detection and the counting results. A typical sample is depicted in Figure 5, which shows a typical predicted result on a raw image. In this sample, the cells with a blue bounding box and label are stained, while the others with a brown bounding box and label are unstained. Based on the predicted results, most cells were detected accurately and categorically divided into two classes: stained (blue ones) and unstained (brown ones). The specific counting results of this sample are shown in Table 6.

**Figure 6:**
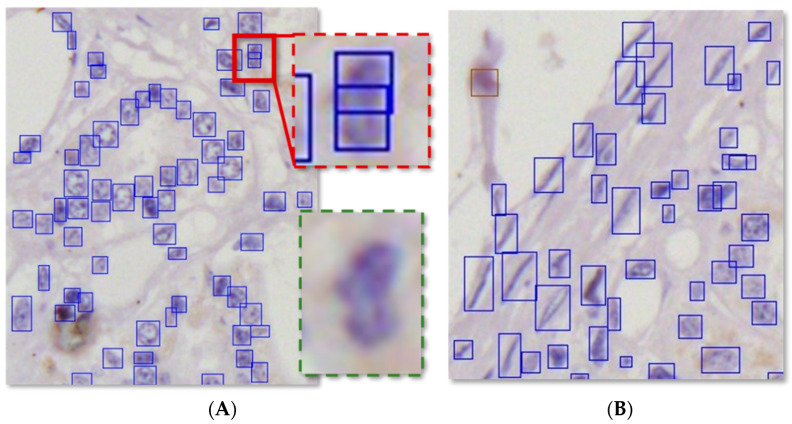
DCRNet’s capabilities to detect (**A**) small-sized adherent cells and (**B**) deformed cells.

It was found that the unstained rates separately output by DCRNet and manual operation were close. This is due to the DCRNet’s powerful feature extraction capability for small-sized objects as well as its adaptive learning and detection capabilities for deformable objects, as shown in Figure 6. In order to make the results more intuitive to facilitate discussion, the labels in the predicted results are removed while only the bounding boxes are retained.

Focusing on Figure 6A, there are quite a few adherent cells in a specific zone. For those adherent cells, DCRNet succeeded in detecting them separately. In the red solid box, there are three cells. Compared with other cells, these three cells are small-sized and severely adherent. The magnified image of the red solid box is located within the red dashed box, and the raw unprocessed image of this area is located within the green dashed box. There are three cells in the red solid box which were all detected by DCRNet. In Figure 6B, there are several cells that have deformed into spindle-shaped or bar-shaped cells due to extrusion. The morphology of these cells is vastly different from other common cells that are round or oval, which makes it difficult to detect them using traditional deep learning models. Instead, DCRNet is capable of detecting deformed cells.

## 4. Discussion

DCRNet has a better performance than other mainstream models, especially in detecting small-sized and extremely deformed cells. However, there is still room for DCRNet to improve in detecting and separating stains from the complicated adhesion of spindle-shaped and bar-shaped cells.

**Figure 7:**
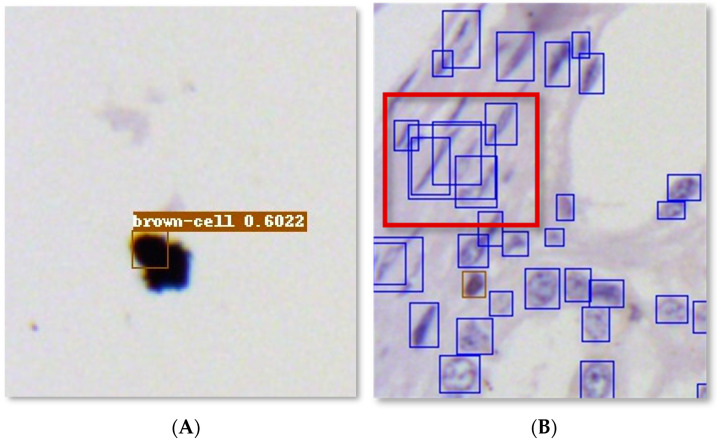
Illustration of (**A**) false positive results and (**B**) invalid boxes of DCRNet.

The counting results of the cells obtained by DCRNet were always higher than the results obtained by manual operation. The authors tried to figure out the cause of this problem. During the process of finding the cause, the following details attracted our attention, as shown in Figure 7.

Figure 7A shows a traditional problem in the deep learning field: the false positive question. The black object which was recognized as a cell is an unspecific stain commonly seen in immunohistochemistry. The deep learning model was confused because the stain is similar to the cells in morphology. DCRNet recognized the stain as a cell but gave a low confidence. However, some true cells with strange shapes were also recognized and given a low confidence. Hence, simply raising the confidence threshold would filter out true cells as well as stains. For Figure 7B, there are some invalid bounding boxes in the red solid box. The reason for this phenomenon is that the cells in the red solid box are extremely deformed and have a certain degree of adhesion. For the adhesion of common round or oval cells, DCRNet can detect cells separately through edge information such as concave points. But for the adhesion of spindle-shaped or bar-shaped cells, the edge information including concave points becomes blurred and difficult to find. Thus, adhesive spindle-shaped and bar-shaped cells, especially the cells with adhesion at their ends, are arduous to detect separately.

In further research, DCRNet can be modified in two ways: (1) enhancing its feature extraction capability for cell interior texture to filter out the stains; (2) enhancing its edge information identification capability for adhesive spindle-shaped and bar-shaped cells, especially cells which are adhesive at their ends, so as to detect and recognize these cells separately.

## 5. Conclusions

This study presented a new deep learning architecture named DCRNet which can be used as a backbone in cell recognition. DCRNet provides automated solutions for the unstained rate of cells via cell counting, which is a compelling reference to evaluate meat quality. DCRNet also frees the labor force from the repetitive and time-consuming counting work. The experimental results show that DCRNet achieved an Average Precision of 81.2% and performed better than traditional neural networks. The unstained rates obtained by DCRNet were less than 0.5% different from those obtained by a pathology experimentalist. All predictions generated by DCRNet can be exported to other image or data analysis software. Moreover, for more accurate cell detection, there is a need to enhance its feature extraction capability for cell interior texture and further explore the identification method of extremely deformed and clustered cells.

## Figures and Tables

**Table 1:** Microscope camera parameters for imaging.

Parameters	Value
Exposure	1/1400
Plotting scale	4080 × 3072
Resolution	variable

**Table 2:** The number of images in each set.

Resolution (Pixel)	Training Set	Validation Set
3840 × 2880 (original images)	135	67
2560 × 1920 (original images)	126	63
1280 × 960	1719	855
640 × 480	6876	3420
320 × 240	27,504	13,680
Total	36,360	18,085

**Table 3:** Results of ablation experiments.

Models	Aggregated Residual Block	New Down-Sampling Block	Deformable Convolution	AP	AP_50_	AP_75_	AP_S_	AP_M_	AP_L_
DCRNet	√	√	√	81.2	99	95.6	80.8	90.3	81.2
DCRNet-v2	√	√		77.8	99.0	94.5	77.6	83.7	76.3
DCRNet-v3	√			75.1	97.3	92.4	74	80.9	75.2
ResNet-101				73.8	96	88.8	73.6	84.5	73.8

Note: “√” means the module is used.

**Table 4:** Prediction results of other mainstream models on testing set.

Model	Backbone	AP	AP_50_	AP_75_	AP_S_	AP_M_	FPS (RTX3090)
Faster R-CNN	DCRNet	81.2	99	95.6	80.8	90.3	12
Faster R-CNN	ResNet-50	69.5	94.6	83	69.2	79.9	9
Faster R-CNN	ResNet-101	73.8	96	88.8	73.6	84.5	7
SSD	VGG-16	43.1	81	42.9	42.7	57.5	38
Yolov3	Darknet-53	49.4	84	54.2	49.2	60	30
Yolov3	ResNet-50	47.3	82.4	53.1	47.4	50.6	28

**Table 5:** Counting results of DCRNet method and manual operation.

	Unstained	Total	Unstained Rate *
DCRNet method	242	1272	0.19
Manual operation	231	1196	0.193

* unstained rate = unstained/total.

**Table 6:** Counting results of typical sample.

	Unstained	Total	Unstained Rate *
DCRNet method	82	809	0.101
Manual operation	68	709	0.096

* unstained rate = unstained/total.

## Data Availability

The original contributions presented in this study are included in the article; further inquiries can be directed to the corresponding authors.
